# Nutritional and Sensory Evaluation of Yoghurt Incorporated with Unripe False Horn Plantain (*Musa paradisiaca* var. “apentu”)

**DOI:** 10.1155/2023/2221302

**Published:** 2023-12-14

**Authors:** Bernadine Olivia Leeward, Francis Alemawor, Godwin Deku

**Affiliations:** ^1^Department of Food Science and Technology, Faculty of Biosciences, College of Science, Kwame Nkrumah University of Science and Technology (KNUST), Ghana; ^2^Dairy/Beef Cattle Research Station, Department of Animal Science, C.A.N.R., Kwame Nkrumah University of Science and Technology (KNUST), Ghana

## Abstract

Unripe plantain (*Musa paradisiaca* L.) is rich in nutrients including minerals, vitamin C, and carbohydrates particularly resistant starches with prebiotic properties. However, the fruit is challenged with limited utilisation, and this contributes to its high postharvest losses along the production and supply chain. Information is lacking on incorporating plantain (UPF) in functional dairy food product development. In a completely randomized design, the study evaluated the effect of unripe false horn plantain (var. “apentu”) flour (UPF) incorporation (*w*/*v*), at 0% (control), 2%, 4%, and 6%, on the composition and sensory quality of yoghurt. The results showed that higher UPF percent incorporation resulted in yoghurts having lower moisture and higher total solid values as well as enhanced nutritional values, in terms of protein, zinc, potassium, calcium, and vitamin C (*P* < 0.05). Mean pH and total titratable acidity values of the yoghurt products were in the ranges of 3.40-3.65 and 1.00-130%, respectively. Conversely, an increase in UPF incorporation generally reduced consumer likeness scores for yoghurt sensory characteristics including appearance, texture, flavour, taste, aftertaste, and overall acceptability. The control AZ product received the highest ratings in all sensory attributes evaluated. Compared with the control AZ, the BX (2% *w*/*w* UPF) yoghurt showed better nutritional quality as well as had comparable ratings for the sensory attributes, particularly in terms of appearance, texture, and flavour. Thus, the formulation containing 2% UPF has the best potential for the production of value-added functional yoghurt, which will be acceptable. However, for high acceptability, further research is needed to improve the impact of UPF incorporation on the overall sensory quality of yoghurt. The study suggests that UPF can serve as a potential supplement for improving the value of yoghurt, and this also contributes to reducing postharvest losses of plantain as a key food security resource. Also, the study findings contribute baseline information to guide future research on functional dairy product development with unripe plantain.

## 1. Introduction

Consumer interest or demand for value-added foods having both enhanced nutritional value and bioactive or health-promoting properties has increased in recent times. Eating such foods, described as functional foods, has the potential to reduce health risk conditions [1]. Prebiotics and probiotics are important functional or health-promoting food supplements or ingredients that encourage beneficial gut microbiota to flourish and provide vital functions that are essential for health [2–4]. Probiotics are live microorganisms (generally LAB) that, when administered in adequate amounts, confer beneficial health effects on the host. Prebiotics are nondigestible food ingredients (including fructo-oligosaccharides, xylo-oligosaccharides, galacto-oligosaccharides, inulin, and resistant starch) that beneficially affect the host by selectively stimulating the growth and/or activity of one or a limited number of certain indigenous bacteria in the colon that can improve the host health. Probiotic food systems, particularly fermented foods, are common. However, the health-promoting properties of probiotic supplements are effectively harnessed when consumed together as this encourages the growth, activities, and a good balance of beneficial gut microbiota/microbiome, to promote health.

Food systems that contain a synergetic mix or combination of probiotics and prebiotics (also referred to as synbiotics) have been strongly advocated or recommended to be one of the effective therapeutic strategies that will give better gut health for the host than those having only probiotics or prebiotics [5]. The efficient implantation of probiotics in colonic microbiota is favoured by synbiotics, as the prebiotics have a stimulating effect on the growth, activities, and stability of probiotic microbiota in the colon which can provide protective effects against colonic carcinogenesis. Also, the prebiotics in synbiotics improve the protection for the survival and viability of orally administered probiotics in the upper gastrointestinal tract [6, 7].

Conventional yoghurt is a common fermented dairy food product enjoyed/consumed by most people globally for its unique flavour, nutrients, and, if unpasteurised, live probiotic LABs (including *Lactobacillus bulgaricus* and *Streptococcus thermophilus*). Prebiotics are usually absent in conventional yoghurt. Exploring the inclusion of natural sources of prebiotics could enhance the functionality and health-promoting value of yoghurt.

Ghana is one of the world's largest producers of plantain (*Musa paradisiaca* L.), recording production levels of 4.72 and 4.7 million tonnes in 2021 and 2022, respectively [8–10]. Varieties available in Ghana include the false horn (“apentu”), French horn (“apem”), and true horn (“asamienu”), and they are commonly enjoyed boiled or fried, whether it is in the ripe or mature-green (unripe) state. Unripe plantain is not only nutritious (containing good amounts of nutrients, including vitamin C, folate, iron, magnesium, potassium, phosphorus, and provitamin A carotenoids) but also a rich source of resistant starches with prebiotic properties [11–17]. Health benefits of unripe plantain include antiulcerogenic properties and ulcer healing activity as well as control of blood pressure and gut health and blood sugar in diabetics due to its low glycemic index, high resistant starch content, and low digestion rate [15–29]. The fermentation of retrograded starch in the human colon leads to the production of small molecular weight fatty acids such as butyric acid that can be absorbed, resulting in the reduction of cholesterol levels in the blood [29–32]. The crystallinity of resistant starch that occurs in native starch granules of plantain makes it less susceptible to hydrolysis [33]. This property makes the resistant starch function as a prebiotic, promoting the growth of beneficial bacteria to impart positive effects on the human colon by alleviating colon diseases such as cancer [34, 35]. Thus, the above information demonstrates the potential of unripe plantain as a functional ingredient worth harnessing in food formulation/development. Previous studies show the application of unripe plantain flour (UPF) in food such as bakery products, snacks, and pasta [12, 17, 36–38].

Currently, there is a dearth of information on the characteristics of functional synbiotic dairy foods involving unripe plantain. This study sought to evaluate the effect of unripe false horn (var. “apentu”) plantain flour (UPF) incorporation/supplementation on the composition and consumer acceptance of yoghurt. In line with SDGs 2 and 12, UPF incorporation in fermented dairy foods would not only create a new healthier yoghurt variety to complement the existing conventional probiotic yoghurt but also contribute to efforts to improve the value and utilisation of plantain, thus reducing its current high postharvest losses along production and supply chains.

## 2. Materials and Methods

### 2.1. Sources of Materials

Unripe false horn plantain (*Musa paradisiaca* var. “apentu”, AAB group), LP™ spray dried whole milk powder containing 26% fat (LacPatrick Dairies Ltd, Northern Ireland), yoghurt starter culture, and 300 mL plastic packaging bowls with lids were purchased from Adum market, Kumasi City, Ghana.

### 2.2. Preparation of Unripe Plantain Flour (Figure 1)

Unripe plantain flour (UPF) was prepared according to protocol by Fagbemi [39] with minor modifications and in a clean environment. Four (4) firm mature-green (stage 1, Adi et al. [40]) plantain fingers (of average fresh weight of 434 g per finger) were washed thoroughly and peeled. A clean sharp stainless knife was used to cut the peeled plantain into slices (2 mm thickness) and water-blanched at 100°C for 10 min and allowed to cool. The blanched slices were evenly spread on cleaned trays and dried in a forced air drying oven (DHG-9240A, Bluepard Instruments, Shanghai, China) at 67°C for 18 h to attain a constant weight. Using a KIMATSU mixer grinder (SPECTRA 750 W, India) at speed 3, the dried slices were milled to pass through a 425 *μ*m sieve (ASTM E11 Standard, No. 40, USA). The obtained unripe plantain flour (UPF) was transferred into a plastic ziplock pouch bag, and after pressing out with most of the air in the bag before sealing, the sample was stored at 4°C in the refrigerator until needed. Average %moisture of UPF was 4.86 (Table 1).

### 2.3. Study Design and Preparation of Set Yoghurt Formulations

The study adopted a completely randomized design to investigate set yoghurt formulations incorporated with UPF at 0% (control), 2%, 4%, and 6% (*w*/*v*). These chosen incorporation levels were informed by a previous work by Batista et al. [41], who incorporated green banana flour in yoghurt, and also based on our preliminary trial work. To assure safe products for sensory evaluation, the yoghurt formulations were prepared at the commercial yoghurt production unit of the KNUST Dairy/Beef Cattle Research Station, Kumasi, Ghana, where all required GHPs and other aseptic practices were carefully followed under the supervision of the production staff. One litre per formulation was prepared. Briefly, for each formulation, the solid ingredients, i.e., milk powder, UPF, and sugar (according to the proportions as indicated in Table 2), were transferred into a clean graduated bowl and thoroughly mixed. Distilled water was then added to the solid mixture and mixed gently and thoroughly to attain a homogenous mixture (the volume, mL, of the water added was the difference between the volume of the solid ingredient mixture in mL and 1000 mL). Each formulation mixture was pasteurized at 72°C for 15 s and cooled to 43-45°C. Subsequently, the same amount of flavour (banana essence) and commercial yoghurt starter culture (at 3%, i.e., 30 mL per 1000 mL) was aseptically added and mixed gently. The packaging bowls were filled with the inoculated mixture and covered, labelled, and incubated in a fermentation unit at 43°C for 4 h. After incubation, the formulated samples were kept in the refrigerator (4°C) before analyses. Each analysis was conducted in triplicate.

### 2.4. Proximate/Physicochemical Properties of Samples

The UPF was analyzed for proximate composition and other physicochemical properties. Proximate composition was determined using AOAC [42] standard methods: moisture content was determined using the oven drying method; crude protein was measured by the Kjeldahl procedure using *N* × 6.25; crude fibre was determined as the fraction remaining after digestion with standard solutions of H_2_SO_4_ and NaOH boiling under controlled heating; crude fat was determined by extraction with petroleum ether in a Soxhlet apparatus; ash was determined by incinerating at 550°C to burn all carbonaceous matter and then weighing the resultant gray ash; total carbohydrate was determined as the difference between 100 and the sum of values for moisture, protein, fat, and ash. Concentrations of potassium, zinc, and calcium were also determined by atomic absorption spectrophotometry according to AOAC [42] standard method. Other physicochemical parameters analyzed include the following: the pH was determined using the ST3100 pH meter (OHAUS Corporation, USA); total titratable acidity was determined as described by Rashmikant [43]; vitamin C was analyzed following the protocol described by Suntornsuk et al. [44]; total soluble solids or °Brix was determined using a digital SOONDA® refractometer 0-85%; total phenolic content was determined as mg gallic acid equivalent per gram (mgGAE/g) using the Folin-Ciocalteu method [45]; total carotenoids were determined by according to the method described by Maclachlan and Zalik [46]. Energy or the caloric value was computed using the Atwater general factor system: carbohydrate (4 kcal g^−1^), lipid (9 kcal g^−1^), and protein (4 kcal g^−1^). Colour analysis was performed using a Konica Minolta Inc. Chroma Meter CR-410 (Japan) to determine the parameters *L*^∗^ (lightness), *a*^∗^ (red/green intensity), and *b*^∗^ (yellow/blue intensity) per the CIE-Lab system (Commission Internationale de l'Eclairage). The yoghurt products were analyzed for moisture, total solids, ash, protein, fat, and vitamin C. All analyses were done in duplicate and reported on an “as is” basis.

### 2.5. Sensory Evaluation of Yoghurt Products

Sensory attributes of yoghurt formulations were evaluated in a consumer acceptance study. Thirty (30) university students (19-31 years) who are regular consumers of yoghurt or fermented milk products with no allergic reaction to milk and have expressed willingness/consent and interest to participate in the test as assessors or panelists were randomly recruited. Also, the number of assessors used was informed by a recommended range of 25-75 panelists for consumer acceptance testing during the product development and optimization [47, 48]. Samples (approximately 30 mL) of the refrigerated yoghurt formulations in identical tasting containers, each coded with a different 3-digit random number, were placed on a tray and served to the assessors. To determine their degree of liking for the products, the evaluators assessed the acceptability of the yoghurt products in terms of appearance, texture, taste, aftertaste, flavour, and overall acceptability using a structured 7-point hedonic scale ranking method, where 1 meant “dislike very much” and 7 “like very much” [49]. Plain water was provided for panelists to rinse their mouth/palate before testing each sample, and standard fluorescent light was used during the evaluation. The study also ensured that the anonymity/confidentiality of the sensory evaluation participants/panelists as well as their evaluation data/information collected was maintained by ensuring that the scorecards used for the sensory evaluation had no request for the names or any personal identifiers of the assessors. The recorded values from the panelists' scorecards were averaged, per each sensory attribute assessed, and reported.

### 2.6. Statistical Analysis

All data collected from analysis of the assessment of yoghurt formulations were expressed as mean ± standard deviation. The data were also subjected to a one-way analysis of variance (ANOVA), and the means were separated by Tukey's multiple comparison test at a 95% confidence level using the Minitab statistical software package (Minitab® version 21.2 (64-bit) ©Minitab LLC, 2022).

## 3. Results and Discussion

### 3.1. Characterization of Unripe Plantain Flour (UPF)

Table 1 shows the physicochemical composition/properties of the UPF produced and used for the preparation of yoghurt formulations. Total soluble sugars were very low while total carbohydrates/starch was the significant component of UPF recorded (83.27%), which agrees with similarly high total carbohydrates/starch of 85.14% and 85.3% reported by Almanza-Benitez et al. [50] and Gutiérrez et al. [51], respectively. About 40-55% of UPF total carbohydrates/starch is resistant starch [12, 50], and this component largely contributes to UPF's prebiotic functionality.

The addition of prebiotics, including resistant starch, FOS, and inulin, to yoghurt has been reported to cause an increase in apparent viscosity and hardness values and a decrease in syneresis of obtained bioyoghurts [52, 53]. Prebiotics added are the water-structuring agents, hence act as thickeners and can form H-bridge complexes with the protein aggregates in the yoghurt. Also, prebiotics such as FOS and inulin added to bioyoghurt exhibited a stimulatory effect on the growth of the probiotics, *Lb. acidophilus*, *Bifidobacterium* sp. growth, and *Str. thermophilus* yoghurts [53].

Our values for other proximate/physicochemical parameters of UPF were either similar or widely different from previous findings. Almanza-Benitez et al. [50] reported 5.48% moisture, 2.76% ash, 0.66% fat, 3.01% protein, and total polyphenols as 5.61 mgGAE/g, for UPF, while Gutiérrez et al. [51] recorded 9.3% moisture, 2.41% ash, 0.37% fat, 2.62% protein, 0.297% crude fibre, and <1.3 °Brix total soluble solids, for UPF.

**Figure 1:**
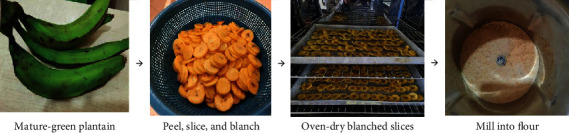
Summary of processing of mature-green plantain pulp into flour (UPF).

Colour is an important consideration in flour quality assessment. Table 1 shows the measured mean values of the colour parameters (*L*^∗^, *a*^∗^, and *b*^∗^ in the CIELAB range) recorded for UPF. Values for colorimetric parameters of a material are influenced by the level of natural pigments, proteins, and fibres and the presence of impurities in the material [54]. The *L*^∗^ value of 78.51 ± 0.55 reflects the lightness or a bright colour of UPF. Although the value (0.01 ± 0.00) recorded for the *a*^∗^ (redness) value parameter is positive, it is near zero indicating no domination of red over green colour. The UPF sample had a *b*^∗^ (yellowness) value (30.04 ± 0.66) much higher than the *a*^∗^ value; the positive *b*^∗^ value is indicative of its yellow colour (Figure 1), which is largely contributed by the natural presence of carotenoids in UPF (Table 1). Gutiérrez et al. [51] also recorded similar colour values of *L*^∗^ = 88.77 ± 0.01, *a*^∗^ = 1.51 ± 0.01, and *b*^∗^ = 17.11 ± 0.01 for native plantain flour. Moreover, the mineral data show that UPF has high levels of K, Fe, and Ca and an adequate amount of Zn.

### 3.2. Effect of UPF Incorporation on Physicochemical/Proximate Composition of Yoghurt Products

The addition of solids influences the physicochemical properties (such as water holding capacity and total solids) and texture/rheological characteristics including viscosity, cohesiveness, firmness or softness, and other sensory properties of a beverage product. Generally, the moisture content of the yoghurt samples decreased as the percent UPF incorporation increased (*P* = 0.004). Moisture content varied in the 78.87-83.29% range, with the highest observed in product AZ (control) and DS yoghurt with 6% *w*/*v* UPF supplementation recording the least moisture value. However, moisture values of products BX and CW were similar to that of product AZ (*P* > 0.05) (Table 3). Ul Haq et al. [55] observed a reduction in the moisture of yoghurt samples with the increase in the lentil flour supplementation (at 10-40%) with moistures in the range of 75.75–65.49%. Iyasele and Ogbeifun [56] recorded higher percent moisture contents of 83.50, 81.80, and 79.50 for yoghurt samples incorporated with 5%, 10%, and 15% plantain flour, respectively.

UPF contains carbohydrate or starch molecules with hygroscopic properties that can interact with water molecules. Thus, a higher percent UPF supplementation reduces the water content per unit amount of the yoghurt product. Güler-Akin et al. [57] observed that cellulose fibre addition significantly influenced the overall properties of bioyoghurt, with a positive effect on the physical and textural properties of the yoghurt, such as water holding capacity, serum separation, viscosity, firmness, adhesiveness, cohesiveness, springiness, gumminess, and chewiness.

Total solids indicate the total amount of solids present in a sample, and they influence product texture/contribute to the thickness or textural properties of yoghurt. The mean total solids recorded for the yoghurt samples ranged from 15.90% to 21.36%, with the value for product DS significantly higher than those recorded for the other products (*P* < 0.05). High solids/dry matter of UPF largely contributed to the observed increase in total solids of yoghurt with an increase in percent incorporation (Table 3). Total solids increase the thickness and firmness of yoghurt, giving yoghurt a custard-like body or texture [58]. Batista et al. [41] recorded average total solids of 14.56% and 19.98%, respectively, for yoghurt samples with 0% and 5% green banana flour. Olugbuyiro and Oseh [58] also reported values ranging from 13.70% to 20.10% as total solids of different yoghurt samples in Nigeria.

Ash content essentially indicates the inorganic bulk of food material, and it is linked to mineral element composition. Mean ash values for yoghurt products varied in a range from 0.53% to 0.64% (*P* = 0.031). However, products BX and CW recorded mean values similar to that of the control AZ (*P* > 0.05). These values were relatively lower compared to the values of ash reported by Iyasele and Ogbeifun [56] but higher compared to the ash content of yoghurt according to the report of Olugbuyiro and Oseh [58]. The variation in ash is largely due to mineral contribution from the UPF. The flesh/pulp of raw plantain contains various mineral elements with an ash content of 0.92%, i.e., on a fresh weight basis [11, 59]. Also, Anajekwu et al. [60] recorded ash values in the range of 2.01–3.69% for flours produced from the pulps of four varieties of unripe plantain. Factors that can influence the mineral concentration of unripe plantain include the level and type of mineral elements in the cultivation soil as well as the variety and mineral absorption capacity of the plantain.

Also, the mean protein content of the yoghurt products increased with UPF incorporation level from 7.23% for AZ (control) to 10.62% for DS (*P* = 0.262) (Table 3). Proteins in the UPF added and cell proteins from the growth of the culture contributed to the observed changes in protein concentration of yoghurt products (Table 2) [11, 53, 61]. Protein values recorded in the present study were relatively higher than those reported by Batista et al. [41] who studied yoghurt incorporated with green banana flour. Yoghurt supplementation with an increase in the total solids, especially protein content, results in stronger texture and less whey separation [62, 63].

Fat analysis carried out for the yoghurt products gave inconsistent results, and those values could not be reported. However, the amount of fat in yoghurt depends on the type of milk and other ingredients used for its preparation, and UPF has some amount of fat (Table 1). UPF and milk powder are essentially the ingredients (Table 2) that account for the fat content of the yoghurt formulations. Thus, the percent fat levels of the yoghurt samples were calculated/estimated based on proportions of UPF and milk powder per 100 mL yoghurt and their respective fat contents, i.e., replicate %fat values for UPF and 26% fat value for milk powder. The estimated fat contents for the yoghurt samples ranged from 2.60 for control AZ to 3.14 for formulation DS, increasing with UPF incorporation level (Table 3). The normal range of fat content for yoghurt is from 0.5% to about 3.5% [64, 65], and the estimated fat values for the yoghurt products (AZ, BX, CW, and DS) fall within this range. The percentage amount of fat in the final yoghurt has a significant effect on the “mouthfeel”; generally, the higher the fat level in the yoghurt, the creamier and smoother it will feel in the consumer's mouth [64].

The mean pH values for UPF-based products (BX, CW, and DS) were lower than those obtained for control AZ (*P* = 0.003). However, increasing the concentration of UPF incorporation only had an insignificant numerical decreasing effect on pH (Table 4). Jenie et al. [66] also observed similar results, where the increase in banana flour in yoghurt preparation decreased the pH. On the other hand, the final mean total titratable acidity (TTA, expressed as %lactic acid) value of yoghurt increased with UPF incorporation level, with average TTA values for UPF-based products (BX, CW, and DS) being significantly different (*P* = 0.006) than that recorded for the control AZ.

The observed increase in acidity or decrease in pH is mainly due to the metabolic activities of probiotic lactic acid bacteria, which break lactose and other sugars in milk and UPF into lactic acid [67]. These observations suggest the prebiotic potential of UPF promoting or enhancing the growth and acid-producing activities of the yoghurt culture. The acidity of yoghurts has been reported to correspond with bacteria activity and was the highest for products with the 2% and 3% resistant starch addition [53]. UPF has a significant amount of resistant/retrograded starch, and this encourages the growth of friendly bacteria such as *Bifidobacterium* and *Lactobacillus*, which act as prebiotics [35, 68]. Increased growth probably resulted in the production of lactic acid and other organic acids, which contributed largely to the observed increase in titratable acidity or pH decrease for the UPF-based yoghurt products. The TTA values obtained for the products fall within the CODEX STAN 243-2003 specifications min. 0.6% (*w*/*w*) for yoghurt [69].

The mean vitamin C content (mg/100 g) for the products varied within the range of 10.75–12.44, marginally increasing with an increase in UPF concentration (*P* = 0.870) (Table 4). These observed increases in vitamin C levels of the yoghurt products are largely attributed to the vitamin C present in the incorporated UPF ingredient (Table 2). Similar results were reported when plantain flour was added to *fura* powder (a millet product) as it increased from 5.0 (mg/100 g) to 9 (mg/100 g) for the control to 40% addition of the plantain flour to the *fura* powder [70]. Raw unripe plantain pulp or flesh contains 20.12 (mg/100 g) vitamin C [11]. Vitamin C is an essential vitamin needed by the body for various metabolic functions, including acting as an antioxidant, enhancing iron absorption, and subsequently fighting against iron deficiency anaemia [71].

### 3.3. Effect of UPF Incorporation on Concentrations of Some Minerals in Yoghurt Products

Mineral elements are essential components of nutrition, basically supplied in balanced diets, and they perform structural, physiological, and metabolic functions in the human body [59]. The levels of zinc, potassium, and calcium found in the yoghurt products were in the following respective ranges (mg/100 g): 6.29–10.89, 67.95–78.84, and 230.25–500.10 (Table 5). These mineral levels recorded were comparable to the findings of previous studies. Also, it was observed that the mean concentrations for the minerals Zn, Ca, and K in the yoghurt products increased with an increase in % UPF incorporation or supplementation. From Table 2, the mineral analysis data show that UPF recorded significant levels for K, Ca, Fe, and Zn, and it contributed to the observed increments for the quantified minerals in the supplemented yoghurt products. This suggests that UPF can serve as a good natural source of minerals to enhance the nutritional value of dairy products like yoghurt. Plantains are rich sources of minerals including Ca, K, and Zn [11, 61].

Zinc is a micronutrient that takes part in the metabolism of proteins, carbohydrates, nucleic acid, and lipids using enzymes. The recommended dietary allowance (RDA) of Zn for adults (19+ years) is 11 mg a day for men and 8 mg for women, with pregnancy and lactation requiring slightly more at 11 mg and 12 mg, respectively [72]. Also, the tolerable upper intake level or the maximum daily intake for Zn that is unlikely to cause harmful effects on health for males and females varies with age categories, and they are as follows: 12 mg for 4-8 years, 23 mg for 9-13 years, 4 mg for 14-18 years, and 40 mg for 19+ years [72]. Our yoghurt products recorded Zn levels between 6 mg and 11 mg. Kibui et al. [73] reported 6.25 to 8.33 mg/100 g as zinc content of yoghurt enriched with chia. Differences in the nutritional status of cows affect mineral levels of milk, which is used for yoghurt making.

Product AZ (control) contained a potassium content of 67 mg/100 g. For the potassium content of plain yoghurts, Amellal-Chibane and Benamara [74] reported an amount of 54.1 mg/100 g while Kibui et al. [73] recorded values within the range of 69–74 mg/100 g. Potassium is useful in nerve functions as well as in countering the negative effects of sodium. Potassium is essential for maintaining the cell and body fluids that control your heart rate and blood pressure [75, 76]. The recommended daily allowance (RDA) of potassium for adults is 4700 mg [77].

Calcium contents (mg/100 g) of 195.0, 800.7, and 703 have been previously reported for plain yoghurts [73, 74, 78]. The calcium content of unripe plantain flour that has undergone some form of fermentation is approximately 77 mg/100 g [61] while that of the boiled unripe plantain pulp and unripe plantain peels contains a calcium content of 59.40 mg/kg and 181 mg/kg, respectively [79]. Cultivation conditions and location influence the calcium levels of plantain [73]. Calcium contributes significantly to optimal bone growth and development as well as the proper functioning of the heart, muscular, and nervous systems [59]. The current RDA for calcium depends on age and sex [80]; RDA of 1300 mg is for the age group of 9-18 years (both males and females) while RDAs of 1000 mg and 1200 mg are for male adults of 19-70 years and >70+ years, respectively, and RDAs of 1000 mg and 1200 mg are for female adults of 19-50 years and 51-70 years, respectively. The recommended upper limit for calcium is 2,500 mg a day for adults 19 to 50. For those 51 and older, the limit is 2,000 mg a day.

### 3.4. Yoghurt Products' Contribution to Nutrients' Daily Values

The nutritional load of food influences the health of a consumer. The Daily Values (DVs) on a nutrient label are reference or recommended amounts (expressed in grams, milligrams, or micrograms) of nutrients to consume or not to exceed each day. DVs are based on a 2,000-calorie diet for healthy adults [81]. The % daily value (%DV) is the percentage of the DV for each nutrient in a serving of the food. This parameter shows how much a nutrient in a serving of food contributes to one's total daily diet, and thus, it helps a person to determine if a serving of food is high or low in nutrient [82]. The general guide to %DV is that 5% DV or less of a nutrient per serving is considered low (a consumer should aim low for total fat, saturated fat, trans fat, cholesterol, and sodium), and 20% DV or more of a nutrient per serving is considered high (a consumer should aim high for vitamins, minerals, and fibre).

Table 6 shows the %DVs for some analyzed nutrients in the yoghurt products. To determine the %DV of nutrient contribution from the yoghurt products, a serving size of 100 g was assumed for the products. Compared with that for the control AZ, the UPF incorporation either maintained or increased %DVs for calcium, potassium, zinc, protein, and vitamin C, in the yoghurt products. If the serving size is increased to 245 g (equiv. of 1 cup), then our results suggest that the formulated yoghurt products are very good sources of Ca, protein, vitamin C, and Zn. However, the products are low sources of potassium.

Although dietary fibre could not be determined in the present study, Garcia-Valle et al. [12] have reported that unripe plantain pulp flour contains about 85.4% total starch, 42.8% resistant starch, and 49.6% dietary fibre. These high values of dietary fibre and resistant starch indicate UPF's potential as a functional ingredient, and thus, the formulated UPF-incorporated composite samples in the present study can be described as functional yoghurt products, enriched with some health properties.

### 3.5. Effect of UPF Incorporation on Sensory Parameters for Yoghurt Products

The effect of the different UPF incorporation levels on consumer acceptability or liking for the yoghurt products was assessed by 30 panelists based on a 7-point hedonic scale (1 = dislike extremely, 7 = like extremely). For the 6 sensory attributes/parameters assessed, mean panelists' scores ranged between 3.10 and 6.07 (Table 7), and the scores decreased as the incorporation level of UPF incorporation in the yoghurt products increased.

Appearance involves characteristics that encompass all visually perceptible sensory impressions of food, and these characteristics include shape, surface, structure, colour, lustre, clarity, cloudiness, and opalescence [83]. The appearance of a food product is an essential quality attribute determined mostly by surface colour, and it is the first sensation that the consumer perceives and uses as a tool to either accept or reject food. Mean preference scores for the appearance of the various yoghurt product treatments generally decreased marginally as the percentage UPF incorporation was increased, and the decreasing order was as follows: BX > AZ > CW > DS (*P* = 0.096). Inherent natural pigments (including the carotenoids) and other physicochemical components or properties of UPF largely influenced the final colour and appearance of the yoghurt products, and these effects impacted panelists' likeness scores for the products.

Usually, yoghurt is often more viscous compared to its starting material milk. Organic acids mainly lactic acid produced during the fermentation lower the pH, which causes coagulation of the milk proteins, and this increases the thickening consistency or texture of the yoghurt products. The incorporation of UPF affected the consumer acceptability score for the texture of yoghurt, following the same decreasing order (*P* = 0.001) as observed for the appearance attribute. However, mean scores for products BX and CW were similar to those of the control product AZ. Some degree of gelatinization of the UPF starches during the heating stage of the mixture may have contributed to the viscous texture of the products; hence, the higher the UPF incorporation percentage, the more viscous the yoghurt becomes [84].

The taste of a food product is the sensation that occurs in the mouth when the food reacts chemically with taste receptor cells located on taste buds, while aftertaste is the taste intensity of a food or beverage that is perceived immediately after that food or beverage is removed (i.e., either swallowed or spat out) from the mouth [85, 86] Panelists' mean scores for the taste and aftertaste attributes of the various yoghurt products followed the decreasing order, AZ > BX > CW > DS as the UPF incorporation level increased (*P* < 0.001), with all the UPF-incorporated yoghurt products being significantly different from the control. The observations made for the likeness ratings for taste and aftertaste attributes of the UPF-supplemented yoghurt products may have been influenced by the UPF's inherent properties. In the unripe state, plantain has a high starch content and low °Brix value (Table 1) which accounts for the low/slight sweet taste of UPF, and increasing the UPF percentage in the yoghurt mixture may have adversely affected the overall taste/aftertaste perception of the products. Also, natural bioactive compounds from UPF, including phenolics, may have influenced the panelists' taste buds; thus, higher UPF percent supplementation impacted negatively on the taste and aftertaste perception ratings for the yoghurt products.

Yoghurt has a unique flavour that is produced as a result of fermentation by lactic acid bacteria. *Staphylococcus thermophilus* is responsible for the formation of yoghurt flavour by the release of lactic acid, acetaldehyde, acetic acid, and diacetyl [84]. The control yoghurt, AZ, had the highest average score for flavour. Acceptance rating for yoghurt flavour decreased as the UPF incorporation level increased (Table 7) (*P* < 0.001). Nonetheless, a marginal difference was observed between the mean flavour scores for product BX and control product AZ (Table 7). UPF chemical constituents, including phenolics, may have interacted or complexed with yoghurt flavour compounds, thus affecting the sensory characteristics of the UPF-based functional yoghurt.

Overall acceptability ratings for the yoghurt products generally decreased (in the order AZ > BX > CW > DS) (Table 7) as the UPF percent supplementation increased, with the 2% UPF-enriched yoghurt recording the next highest mean overall acceptance score after the control AZ (Table 7). Multiple sensory attributes play a significant role in consumer acceptance, and the observed decreasing scores for the overall rating of the products with increasing UPF percent supplementation may have been influenced by the combined consideration of the similar trend observations made for almost all the individual sensory attributes (Table 7).

## 4. Conclusion

The study evaluated the effect of unripe false horn plantain flour (UPF) incorporation/supplementation on yoghurt's composition and sensory acceptability. The study findings indicated that UPF incorporation enhanced the nutritional quality of yoghurt, in terms of protein, zinc, potassium, calcium, and vitamin C. Conversely, an increase in UPF incorporation generally reduced consumer likeness for yoghurt sensory characteristics including appearance, texture, flavour, taste, aftertaste, and overall acceptability. The control AZ product received the highest ratings in all sensory attributes evaluated. Compared with the control AZ, the BX (2% *w*/*w* UPF) yoghurt showed better nutritional quality as well as had comparable ratings for the sensory attributes, particularly in terms of appearance, texture, and flavour. Thus, the formulation containing 2% UPF has the best potential for the production of value-added functional yoghurt, which will be acceptable and recommended. However, for high acceptability, further research is needed to improve the impact of UPF incorporation on the overall sensory quality of yoghurt. The study suggests that UPF can serve as a potential supplement for the value addition of fermented dairy products such as yoghurt, and thus, by this application, the economic value of plantain can be improved. Also, the study demonstrates one of the potential applications to maximise the value of plantain as a key food security resource, and the findings contribute baseline information to guide future research for functional dairy product development with plantain.

## Figures and Tables

**Table 1:** Physicochemical characterization of UPF.

Parameter	Mean ± SD
*Proximate composition (g/100* g)	
Moisture	4.86 ± 0.10
Ash	2.61 ± 0.03
Crude fat	9.02 ± 0.18
Crude fibre	0.55 ± 0.05
Crude protein	0.23 ± 0.01
Total carbohydrates	83.27 ± 0.24
Energy (kcal/100 g)	415.19 ± 0.60
*Minerals (mg/100 g)*	
Zinc	2.80 ± 0.326
Calcium	213.79 ± 145.274
Iron	227.82 ± 10.278
Potassium	9930.35 ± 0.216
*Other physicochemical parameters*	
Total soluble solids (°Brix)	0.60 ± 0.14
pH	4.89 ± 0.08
Titratable acidity (%)	0.06 ± 0.00
Vitamin C (mg/100 g)	47.91 ± 0.14
Total phenolics (mgGAE/g)	0.772 ± 0.0013
Total carotenoids (mg/g)	1.113 ± 0.0198
*Colour parameters*	
*L* ^∗^	78.51 ± 0.55
*a* ^∗^	0.01 ± 0.00
*b* ^∗^	30.04 ± 0.66

**Table 2:** Proportions of unripe plantain flour (UPF), milk, and sugar, for the preparation of 1 L set yoghurt per formulation.

Formulation	UPF (g)	Milk powder (g)	Sugar (g)
AZ (control)	0	100	70
BX (2% *w*/*v*)	20	100	70
CW (4% *w*/*v*)	40	100	70
DS (6% *w*/*v*)	60	100	70

**Table 3:** Effect of UPF incorporation level on some proximate components of yoghurt products.

Product	Parameter (%)				
	Moisture	Total solids	Ash	Protein	Fat^∗^
AZ	84.10 (0.988)^a^	15.90 (0.988)^b^	0.542 (0.0387)^ab^	7.23 (2.290)^a^	2.60 (0.000)^a^
BX	81.33 (1.358)^ab^	18.67 (1.358)^ab^	0.525 (0.0017)^b^	7.89 (0.293)^a^	2.78 (0.004)^b^
CW	81.21 (0.820)^ab^	18.79 (0.820)^ab^	0.554 (0.0251)^ab^	9.74 (0.876)^a^	2.96 (0.007)^c^
DS	78.64 (1.560)^b^	21.36 (1.560)^a^	0.635 (0.0064)^a^	10.62 (2.020)^a^	3.14 (0.011)^d^

^a-d^Mean (SD) values in the same column that share the same (superscript) letter are not significantly different, *P* > 0.05 (means grouped by the Tukey test @ 95% confidence). Yoghurt products (AZ = 0%*w*/*v* UPF (control), BX = 2%*w*/*v* UPF, CW = 4%*w*/*v* UPF, and DS = 6%*w*/*v* UPF). ^∗^Percent mean fat was calculated/estimated based on proportions of UPF and LP™ milk powder per 100 mL yoghurt and their respective fat contents, i.e., replicate %fat values for UPF and 26% fat value for LP™ milk powder.

**Table 4:** Effect of UPF incorporation level on pH, TTA, and vitamin C content of yoghurt products.

Product	Parameter		
	pH	TTA (% lactic acid equiv.)	Vitamin C (mg/100 g)
AZ	3.63 ± 0.081^a^	1.067 ± 0.0302^b^	10.75 ± 0.896^a^
BX	3.41 ± 0.015^b^	1.218 ± 0.0852^a^	11.14 ± 0.945^a^
CW	3.47 ± 0.015^b^	1.220 ± 0.0362^a^	11.96 ± 2.270^a^
DS	3.47 ± 0.045^b^	1.274 ± 0.0361^a^	12.44 ± 3.660^a^

^a,b^Mean ± SD values in the same column that share the same superscript letter are not significantly different, *P* > 0.05 (means grouped by the Tukey test @ 95% confidence). Yoghurt products (AZ = 0%*w*/*v* UPF (control), BX = 2%*w*/*v* UPF, CW = 4%*w*/*v* UPF, and DS = 6%*w*/*v* UPF).

**Table 5:** Concentrations of some minerals in yoghurt products supplemented with varying levels of UPF.

Sample	Mineral (mg/100 g)		
	Zinc (Zn)	Calcium (Ca)	Potassium (K)
AZ	6.29 ± 0.003^a^	230.25 ± 0.098^a^	67.95 ± 0.036^a^
BX	6.35 ± 0.001^b^	319.50 ± 0.079^b^	73.04 ± 0.061^b^
CW	8.85 ± 0.004^c^	329.85 ± 0.113^c^	73.28 ± 0.034^c^
DS	10.87 ± 0.012^d^	500.10 ± 0.100^d^	78.84 ± 0.069^d^

^a-d^Mean (SD) values in the same column that share the same superscript letter are not significantly different, *P* > 0.05 (means grouped by the Tukey test @ 95% confidence). Yoghurt products (AZ = 0%*w*/*v* UPF (control), BX = 2%*w*/*v* UPF, CW = 4%*w*/*v* UPF, and DS = 6%*w*/*v* UPF).

**Table 6:** %DV for some nutrients in the yoghurt products.

Nutrient	Daily value (DV)^1^	%DV for nutrient per product at 100 g serving size			
		AZ	BX	CW	DS
Calcium	1300 mg	18	25	25	39
Potassium	4700 mg	2	2	2	2
Vitamin C	90 mg	12	12	13	14
Zinc	11 mg	57	58	81	99
Protein	50 g	15	16	20	21

^1^[82].

**Table 7:** Sensory evaluation of UPF-incorporated yoghurt products (no. of panelists = 30).

Sample	Parameter					
	Appearance	Texture	Taste	Aftertaste	Flavour	Overall acceptability
AZ	4.93 ± 1.799^a^	4.60 ± 1.812^a^	6.03 ± 0.765^a^	5.80 ± 1.157^a^	6.07 ± 0.944^a^	6.00 ± 0.871^a^
BX	5.27 ± 1.437^a^	4.70 ± 1.643^a^	4.90 ± 1.322^b^	4.67 ± 1.373^b^	5.33 ± 1.269^a^	5.13 ± 1.358^b^
CW	4.50 ± 1.676^a^	3.87 ± 1.634^ab^	3.90 ± 1.242^c^	3.93 ± 1.363^bc^	4.37 ± 1.351^b^	4.23 ± 1.223^c^
DS	4.23 ± 1.870^a^	3.13 ± 1.548^b^	3.70 ± 1.208^c^	3.73 ± 1.112^c^	4.47 ± 1.252^b^	3.83 ± 1.053^c^

^a-c^Values (mean ± SD) in the same column that share the same (superscript) letter are not significantly different, *P* > 0.05 (means grouped by the Tukey test @ 95% confidence). Yoghurt products (AZ = 0%*w*/*v* UPF (control), BX = 2%*w*/*v* UPF, CW = 4%*w*/*v* UPF, and DS = 6%*w*/*v* UPF).

## Data Availability

Data used to support the findings of this study are included within the article.
